# Supplementary material for: Pharmacokinetics and safety after once and twice a day doses of meclizine hydrochloride administered to children with achondroplasia
Source: PLoS One. 2020 Apr 13;15(4):e0229639. doi: 10.1371/journal.pone.0229639 (PMC7153885; doi:10.1371/journal.pone.0229639)
Supplement: S2 Checklist — (PDF) [file pone.0229639.s002.pdf]

軟骨無形成症患者（小児）に対する
塩酸メクリジン製剤の安全性及び薬物動態の検討

治験実施計画書

＜治験責任医師＞
名古屋大学医学部附属病院・整形外科
准教授　鬼頭　浩史
名古屋市昭和区鶴舞町 65
TEL：052-741-2111　FAX：052-744-2260
e-mail：hkitoh@med.nagoya-u.ac.jp

＜治験調整事務局＞
名古屋大学大学院医学系研究科
整形外科
〒466-8560　名古屋市昭和区鶴舞町 65
TEL：052-744-2257　FAX：052-744-2260

治験実施計画書番号：　CAMCR-009
版　番　号　：　第 3.0 版
作成年月日：　2018 年 5 月 31 日

秘密保全のお願い

本治験実施計画書は、治験の直接関係者に限定して提供される情報が含まれています。本治験実施計画書の内容を公表又は第三者へ開示する場合には、事前に治験責任医師の承認を得るようお願いします。

本治験は、医薬品、医療機器等の品質、有効性及び安全性の確保等に関する法律及び医薬品の臨床試験の実施の基準（GCP）に関する省令及び関連法規、治験実施計画書を遵守して行うものとする。

目　　次

治験実施計画書の要約 6

1. 治験実施の経緯及び背景情報 11

2. 治験の目的 13

3. 対象 13

3.1 対象 13

3.2 選択基準 13

3.3 除外基準 13

4. 被験者への説明及び同意 14

4.1 説明文書及び同意文書の作成 14

4.2 説明文書に記載すべき内容 14

4.3 同意取得の方法 15

4.4 同意説明文書の改訂 16

5. 登録 17

5.1 登録の手順 17

5.2 登録に際しての注意事項 17

5.3 本治験の被験者として不適格と判断された場合 17

6. 治験の方法 18

6.1 治験の種類・デザイン 18

6.1.1 治験の種類 18

6.1.2 治験デザイン 18

6.2 投薬方法 18

6.3 評価項目 19

6.3.1 主要評価項目 19

6.3.2 副次的評価項目 19

7. 目標症例数及び治験実施期間 19

7.1 目標症例数 19

7.2 治験実施期間 19

8. 治験薬 20

8.1 治験薬の概要 20

8.2 治験薬の製剤組成 20

8.3 治験薬の包装及び表示 21

8.4 治験薬の取扱い、保管及び管理 21

9. 併用薬・併用療法 21

9.1　併用を禁止・制限する薬剤・療法 21
9.2　併用薬・併用療法の記録 22
10. 安全性評価委員会 22
11. 投薬・検査・観察 23
11.1 投薬・検査・観察スケジュール 23
11.2 検査・観察項目及び時期 25
11.2.1 スクリーニング期間 25
11.2.2 治験薬投与日（朝投与前） 25
11.2.3 治験薬投与日（朝投与１時間後） 26
11.2.4 治験薬投与日（朝投与２時間後） 26
11.2.5 治験薬投与日（朝投与３時間後） 26
11.2.6 治験薬投与日（朝投与４時間後） 26
11.2.7 治験薬投与日（朝投与８時間後） 26
11.2.8 治験薬投与日（朝投与 10 時間後/夕投与前） 26
11.2.9 治験薬投与日（朝投与 11 時間後/夕投与 1 時間後） 27
11.2.10 治験薬投与日（朝投与 12 時間後/夕投与 2 時間後） 27
11.2.11 治験薬投与日（朝投与 13 時間後/夕投与 3 時間後） 27
11.2.12 治験薬投与日（朝投与 14 時間後/夕投与 4 時間後） 27
11.2.13 治験薬投与２日目（朝投与 24 時間後） 27
11.2.14 治験薬投与終了 1 週間後 28
11.2.15 中止時 28
12. 有害事象 28
12.1 有害事象等の定義 28
12.2 有害事象の評価及び基準 29
12.2.1 発現日 29
12.2.2 程度 29
12.2.3 重篤性 29
12.2.4 処置の有無 29
12.2.5 処置内容 29
12.2.6 治験薬との因果関係 30
12.2.7 転帰 30
12.2.8 転帰日 30
12.3 有害事象の追跡 30
12.4 有害事象の記録事項 30
13. 被験者の安全性の確保 31
13.1 予想される有害事象 31

13.1.1 治験薬に関連する有害事象 31
13.2 重篤な有害事象発現時の対応 32
13.3 被験者の他の主治医への連絡等 32
14. 被験者の中止基準及びその手順 32
14.1 被験者の中止基準 32
14.2 中止の手順 33
15. 解析 33
15.1 対象集団 33
15.1.1 薬物動態の解析対象集団 33
15.1.2 薬物蓄積性の解析対象集団 34
15.1.3 安全性の解析対象集団 34
15.2 解析計画 34
15.2.1 薬物動態評価項目 34
15.2.2 蓄積性評価項目 35
15.2.3 安全性評価項目 35
15.3 統計解析計画書の変更 35
15.4 データの取り扱い 35
16. 治験実施計画書の遵守、逸脱及び改訂 36
16.1 治験実施計画書の遵守 36
16.2 治験実施計画書からの逸脱 36
16.3 治験実施計画書の改訂 36
17. 治験の中止又は中断 37
18. CRF に関する事項 37
19. 原資料等の直接閲覧 38
20. 治験の品質管理及び品質保証 38
21. 倫理 38
21.1 治験の倫理的実施 38
21.2 治験審査委員会 38
21.3 被験者の秘密保全 38
22. 記録等の保存 38
22.1 実施医療機関において保存する記録類 39
22.2 治験責任医師において保存する記録類 39
23. 治験の資金源と利益相反の管理 38
24. 金銭の支払い 40
25. 健康被害に対する補償 40
26. 治験成果の帰属と結果の公表 40

27. 治験の登録と進捗状況の報告.. 41
28. 実施体制.. 41

略号及び用語の定義

略号一覧

略号	省略していない表現
ACH	Achondroplasia（軟骨無形成症）
AUC	Area under the curve (血中濃度曲線下面積)
Cmax	maximum concentration (最高血中濃度)
CNP	C-type Natriuretic Peptide (C 型ナトリウム利尿ペプチド)
CRF	Case Report Form　（症例報告書）
FAS	full analysis set　(最大の解析対象集団)
FGFR3	Fibroblast growth factor receptor 3（線維芽細胞増殖因子受容体 3）
GCP	Good Clinical Practice（医薬品・医療機器等の臨床試験の実施に関する基準)
PPS	per protocol set　(試験計画書遵守集団)
Tmax	Time to reach Cmax　(最高血中濃度到達時間)
t 1/2	Elimination half-life　(血中濃度半減期)
UMIN	University Hospital Medical Information Network（大学病院医療情報ネットワーク）

臨床検査項目の略号一覧

略号	省略していない表現
TP	Total protein（総蛋白）
Alb	Albumin（アルブミン）
ALT	Alanine Aminotransferase(アラニンアミノ基転移酵素)
AST	Aspartate Aminotransferase（アスパラギン酸アミノ基転移酵素）
BUN	Blood urea nitrogen（尿素窒素）
Cl	Chlorine（クロール）
Cre	Creatinine（クレアチニン）
CRP	C-Reactive Protein（C 反応性タンパク）
K	Potassium（カリウム）
Na	Sodium（ナトリウム）

Summary of the protocol

Title	Pharmacokinetics and safety of meclizine hydrochloride administered to achondroplasia children
Purpose of the study	We administer meclizine hydrochloride to achondroplasia children to examine pharmacokinetics for 24 hours after administration as well as drug accumulation one weak after administration, together with safety evaluation.
Trial number	CAMCR-009
Trial design	Unblinded two group trial
Subjects	Achondroplasia patients
Inclusion criteria	Patients who meet all the following conditions<br>① Patients who were diagnosed as achondroplasia one year or more before consent was obtained based on the diagnostic criteria established by the study group of Ministry of Health, Labour, and Welfare<br>② Patients aged from 5 to younger than 11 years at the time of obtaining consent<br>③ Patients who can be hospitalized from the day before administration to the day after administration and are expected to complete this trial during the evaluation period<br>④ Patients whose consent was obtained in writing by a representative<br>⑤ Patients who obtained an ascent in writing when the subject is 7 years of age or older<br>⑥ Patients who can take tablets
Exclusion criteria	Exclude patients who comply with any of the followings<br>① Patients who took a drug containing meclizine hydrochloride within 28 days before administration of meclizine<br>② Patients who underwent limb lengthening within 28 days prior to administration of meclizine or who are scheduled to undergo limb lengthening during the study period<br>③ Patients with serious complications<br>④ Patients whose weight of less than 11 kg<br>⑤ Patients with symptoms of dysutia<br>⑥ Patients who have been diagnosed as glaucoma<br>⑦ Patients who are allergic to meclizine hydrochloride<br>⑧ In addition, patients who were decided inappropriate by the investigator or subinvestigator in conducting the trial
Target number of	12cases（once a day group: 6 cases、twice a day group: 6 cases）

cases	The first 6 subjects are assigned to "once a day administration group", and subsequent 6 subjects are assigned to "twice a day administration group"
Trial drug	Name：Sorashidon (meclizine hydrochloride)
Medication methods	Once a day group：Take meclizine once a day in a fasted condition<br>Twice a day group：Take meclizine twice a day (in the morning and in the evening) in a fed condition
Prohibited drugs and therapies	Concomitant drugs:<br>Following drugs is prohibited from 7 days before administration of meclizine until the end of the evaluation period.<br>① Drugs that have been reported to have the ability to suppress FGFR3 signaling (CNP analogues not approved in Japan, statins etc.)<br>② Drugs such as anti-motion sickness drugs containing meclizine hydrochiloride<br>Following drugs is prohibited within 24 hours before administration of meclizine and within 24 hours after completion of administration.<br>③ common col medicine, antipyretic analgesic, sedative, antitussive expectorant, internal medicine containing antihistamine (internal medicine for rhinitis, medicine for allergy etc.)<br>Following treatments are prohibited during the period from obtaining consent to the end of the evaluation period.<br>④ Patients who were treated with growth hormone at the time of obtaining consent do not discontinue or change treatment until the end of the evaluation period.<br>⑤ limb lengthening surgery
Evaluation items	【Primary endpoints】<br>Safety: Name, extent, and rate of adverse events or side effects<br>pharmacokinetics・accumulation：plasma concentration of meclizine<br>① Cmax (during 24 hours)<br>② Tmax (during 24 hours)<br>③ t 1/2 (during 24 hours)<br>④ AUC (during 24 hours)<br>⑤ Accumulation（7 days after administration of meclizine）<br>【Secondary endpoints】<br>Simulation of plasma concentration of meclizine

	（Simulate pharmacokinetics with repeated administration for 14 days based on the plasma concentration of meclizine after once or twice a day administration）
Evaluation period	8 days (administration period 1 days ＋ follow-up period 7 days）
Principal investigator	Hiroshi Kitoh, Department of Orthopaedic Surgery, Nagoya University Hospital
Procurement of investigational drugs	Purchase a commercial product and attach a label for clinical trail
Conduct medical institution	Department of Orthopaedic Surgery, Nagoya University Hospital
Trail period	From June, 2018 to March, 2019

投与・検査・観察スケジュール

1 日 1 回投与群

項目	同意取得	スクリーニング期間		評価期間													中止時
				1日目											2日目・24時間後	投与終了7日後	
				朝投与													
		スクリーニング	登録	投与前	投与1時間後	投与2時間後	投与3時間後	投与4時間後	投与8時間後	投与10時間後	投与11時間後	投与12時間後	投与13時間後	投与14時間後			
【参考】朝9時投与の場合の時刻				9 AM	10 AM	11 AM	12 AM	1 PM	5 PM	7 PM	8 PM	9 PM	10 PM	11 PM	9 AM		
同意取得	●																
被験者背景調査		●															
既往歴、合併症		●															
登録			●														
治験薬服用				●													
身長		●															
体重		●		●											●	●	●
バイタルサイン		●		●	●	●	●	●	●	●					●	●	●
12誘導心電図		●		●*											●	●	●
胸部レントゲン 検査		●															
血液学的検査		○		●												●	●
血液生化学的検査		○		●												●	●
尿検査		○		●												●	●
薬物濃度測定				●	●	●	●	●	●	●					●	●	●
有害事象					●	●	●	●	●	●	●	●	●	●	●	●	●
併用薬・併用療法		●	●	●	●	●	●	●	●	●	●	●	●	●	●	●	●

○ :投与開始前28日以内に日常診療として検査している場合は、既存資料としての使用について被験者の同意が得られた場合、当該データをスクリーニング時のデータとして使用する。
＊ :スクリーニング検査を治験薬投与日から28日以内に実施している場合には、スクリーニング時のデータを採用する。

１日２回投与群

項目	同意取得	スクリーニング期間：スクリーニング	スクリーニング期間：登録	評価期間 1日目 朝投与：投与前	朝投与：投与1時間後	朝投与：投与2時間後	朝投与：投与3時間後	朝投与：投与4時間後	朝投与：投与8時間後	朝投与：投与10時間後／夕投与：投与前	夕投与：投与1時間後	夕投与：投与2時間後	夕投与：投与3時間後	夕投与：投与4時間後	2日目・24時間後	投与終了7日後	中止時
【参考】朝9時、夕7時投与の場合の時刻				9 AM	10 AM	11 AM	12 AM	1 PM	5 PM	7 PM	8 PM	9 PM	10 PM	11 PM	9 AM		
同意取得	●																
被験者背景調査		●															
既往歴、合併症		●															
登録			●														
治験薬服用				●						●							
身長		●															
体重		●		●											●	●	●
バイタルサイン		●		●	●	●	●	●	●	●	●	●	●	●	●	●	●
12誘導心電図		●		●*											●	●	●
胸部レントゲン検査		●															
血液学的検査		○		●												●	●
血液生化学的検査		○		●												●	●
尿検査		○		●												●	●
薬物濃度測定				●	●	●	●	●	●	●	●	●	●	●	●	●	●
有害事象					●	●	●	●	●	●	●	●	●	●	●	●	●
併用薬・併用療法		●	●	●	●	●	●	●	●	●	●	●	●	●	●	●	●

○ :投与開始前28日以内に日常診療として検査している場合は、既存資料としての使用について被験者の同意が得られた場合、当該データをスクリーニング時のデータとして使用する。
* :スクリーニング検査を治験薬投与日から28日以内に実施している場合には、スクリーニング時のデータを採用する。

1. 治験実施の経緯及び背景情報

軟骨無形成症（achondroplasia: ACH）は、最も頻度の高い四肢短縮型低身長を呈する骨系統疾患で、骨伸長の抑制因子である線維芽細胞増殖因子受容体 3 (fibroblast growth factor receptor 3：FGFR3)の機能獲得型変異による過剰な FGFR3 活性化が原因であり、低身長に加えて脊柱管狭窄症・大後頭孔狭窄などの重篤な合併症も生じる。特に四肢骨の長径成長が障害されることにより成人における最終身長は 120～130cm であり日常生活に多大な支障をきたす。現在、軟骨無形成症における過剰に活性化した FGFR3 シグナルを抑制する根本的治療はなく、低身長に対する対症的な治療として内科的には成長ホルモン治療、外科的には骨延長術が行われているが、前者は有効性に乏しく、後者は侵襲性が高いのが難点である。 近年、FGFR3 シグナルを抑制する複数の低分子化合物が同定されたが、これら化合物は非臨床試験での安全性が担保されていない（J Biol Chem 2010、Hum Mol Genet 2012）。生理活性物質である C 型ナトリウム利尿ペプチド（C-type natriuretic peptide：CNP）は ACH モデル動物において有効性が示されたが、半減期が数分と著しく短い（Nat Med 2004）。また、米国で開発された CNP アナログ（BMN111）は臨床試験を実施中であるが、注射剤であり小児への長期投与は負荷が大きい（Am J Hum Genet 2012）。iPS 細胞を使用したスクリーニング系によりスタチンの FGFR3 シグナル抑制効果が報告されたが、至適使用量や期間、副作用に関する検討はなされていない（Nature 2014）。日常生活に多大な支障を及ぼす ACH に対する治療は、小児期から治療開始が必要となるため、侵襲性が低い根本的治療薬の開発が必要であると考える。

塩酸メクリジンは米国において 1957 年 2 月 14 日に乗り物酔い症状の緩和の効能で承認された処方箋医薬品である。また、本邦においては一般用医薬品（第二種医薬品）として 1981 年から発売されており、3 才以上が適用対象となっている。このように、塩酸メクリジンは先進国において 60 年以上の使用実績があり、この間、日米の薬事規制当局から安全面で規制を加える必要があるとの見解が出されたことはない。

2013 年、名古屋大学大学院医学研究科の鬼頭、松下、大野らは ACH モデル細胞に 1,186 種類の FDA 既承認薬を添加した網羅的薬効スクリーニング法により、塩酸メクリジンが各種軟骨系細胞において、FGFR3 下流の MAPK 経路において Erk のリン酸化を抑制することを見出した。また、胎生期マウス脛骨器官培養系において、メクリジンは骨伸長作用を示した。さらに、成長期の ACH モデルマウスに乗り物酔い防止薬で得られる血漿中濃度の範囲内に相当するメクリジンを混餌経口投与したところ、モデルマウスの骨伸長をレスキューした。単回投与 PK 試験において、マウスでは 2mg/kg 投与で乗り物酔い薬としてのメクリジンの臨床使用量(25mg)と同レベルでの血中濃度が得られ

ることを確認した。そこで定量的に薬剤を経口投与したところ、メクリジン 1mg/kg/day および 2mg/kg/day の 1 日 2 回経口投与により濃度依存性にモデルマウスの骨長が促進した。組織学的にも成長軟骨帯における肥大軟骨細胞層の幅の拡大を認めた。また、メクリジン投与により長管骨や脊椎の骨量が増大するとともに、骨幹端部の骨質の改善も認め、in vivo における FGFR3 抑制作用を確証した。

塩酸メクリジンは国内外で古くから医療用あるいは一般用医薬品として使用されているが、治験実施に利用可能な非臨床安全性試験データは揃っていない。そこで、PMDA 薬事戦略相談の助言を受けて、ラットおよびイヌの単回投与 TK 試験、1 週間および 2 週間反復投与予備毒性試験を実施した。2 週間反復投与試験での無毒性量はラット 15mg/kg (M,F)、イヌ 300mg/kg(M),100mg/kg(F)であり、種差を認めた。追加実施した蛋白結合率・肝代謝固有クリアランス・代謝物プロファイリング試験の結果から、ラットではヒト、イヌで認められない代謝物（二水酸化体）の存在を認め、ラットで観察された毒性がげっ歯類特有のものである可能性が示唆された。さらに、遺伝毒性試験および安全性薬理試験を実施して安全性を確認し、2017 年 3 月までに「医薬品の臨床試験及び製造販売承認申請のための非臨床安全性試験の実施についてのガイダンスについて」（平成 22 年 2 月 19 日付薬食審査発 0219 第 4 号、ICH M3（R2）ガイダンス）に基づいた新規有効成分の臨床第 1 相試験の治験届を提出するのに必要な非臨床安全性試験を完了した。

2017 年 8 月 24 日に PMDA の対面助言を実施し、次のような助言を受けた。
申請区分については、本剤の有効成分が 一般用医薬品として国内で承認・販売されていることを踏まえると、新規有効成分（区分１－（１）)に該当するものではなく、新効能医薬品（区分１－（４）)に該当すると考える。小児 ACH 患者を対象とした単回投与試験は受け入れ可能である。
ICH M3（R2）ガイダンスを踏まえると、幼若動物試験を実施しなくても、1～3 回程度の投与であれば、単回投与試験内で実施することは可能と考える。したがって、今後予定している反復投与試験等において、本剤 50 mg/日（1 回 25mg を 1 日 2 回） の投与も想定しているのであれば、小児 ACH 患者を対象とした単回投与試験において、本剤 25 mg 1 日 2 回投与時における薬物動態及び安全性について検討することも一案と考える。

以上、PMDA の助言を踏まえ、小児 ACH 患者を対象とした塩酸メクリジン 25 mg 錠の 1 日 1 回並びに 1 日 2 回投与時における薬物動態及び安全性を検討する試験を計画した。

2. Purpose

The purpose of this clinical trial is to examine pharmacokinetics and safety after oral administration of meclizine for achondroplasia children.

3. Patients

3.1 patients

achondroplasia patients

3.2 Inclusion criteria

Patients who meet all the following conditions

① Patients who were diagnosed as achondroplasia one year or more before consent was obtained based on the diagnostic criteria established by the study group of Ministry of Health, Labour, and Welfare

② Patients aged from 5 to younger than 11 years at the time of obtaining consent

③ Patients who can be hospitalized from the day before administration to the day after administration and are expected to complete this trial during the evaluation period

④ Patients whose consent was obtained in writing by a representative

⑤ Patients who obtained an ascent in writing when the subject is 7 years of age or older

⑥ Patients who can take tablets

【設定根拠】

① 治療対象となる患者集団として設定した。

② 被験薬の薬理作用は骨端線が閉鎖した場合、期待できない。期待される薬効を得るには骨端線の閉鎖する思春期依以前の投与が必要である。有効性は第 2 相試験で評価する予定であるが、5 歳以上 11 歳未満の軟骨無形成症患者の成長速度は性別、年齢によらず年間 4cm（全国調査に基づいた軟骨無形成症患児の身長の検討）であり、成長曲線との比較により第 2 相試験で有効性評価が可能な患者集団と同一の集団として設定した。

また、被験薬は成人における PK データはあるものの、治療対象となる 5 歳から 11 歳の児童における PK データはない事から、臨床至適用量の推定を行うためには、児童を対象とした薬物濃度推移、動態のデータを取得することが必要である為、対象集団として設定した。

③ 治験計画書に従い治験が実施できる患者として設定した。

④⑤ 被験者からの同意取得を原則とするが、年齢を勘案し、代諾者のみからの取得でも治験参加を可とするため、設定した。

⑦ 治験薬が内服用錠剤であるため設定した。

3.3 Exclusion criteria

Exclude patients who comply with any of the followings

① Patients who took a drug containing meclizine hydrochloride within 28 days before administration of meclizine

② Patients who underwent limb lengthening within 28 days prior to administration of meclizine or who are scheduled to undergo limb lengthening during the study period

③ Patients with serious complications

④ Patients whose weight of less than 11 kg

⑤ Patients with symptoms of dysutia

⑥ Patients who have been diagnosed as glaucoma

⑦ Patients who are allergic to meclizine hydrochloride

⑧ In addition, patients who were decided inappropriate by the investigator or subinvestigator in conducting the trial

【設定根拠】

① 薬剤の安全性、薬物動態を適切に評価するため、設定した。

②③④⑤⑥⑦⑧ 被験者の安全性を確保するために設定した。

4. 被験者への説明及び同意

4.1 説明文書及び同意文書の作成

治験責任医師は、説明文書、アセント文書及び同意文書（以下、同意説明文書という）を作成する。同意説明文書は一体化した文書又は一式の文書とし、必要な場合にはこれを改訂する。

作成した当該文書は実施医療機関の長に提出し、同意取得前に治験審査委員会にて承認を得る。

4.2 説明文書に記載すべき内容

同意説明文書には、少なくとも次の事項が含まれていなければならない。

(1) 治験が研究を伴うこと

(2) 治験の目的

(3) 治験責任医師の氏名、職名及び連絡先

(4) 治験の方法（治験の試験的側面、被験者の選択・除外基準を含む）

(5) 予期される臨床上の利益及び危険性又は不便

(6) 当該被験者に対する他の治療方法の有無及びその治療方法に関して予測される重要な利益及び危険性

(7) 被験者の治験への参加予定期間

(8) 治験への参加は被験者の自由意思によるものであり、被験者又は代諾者は、被験者の治験への参加を随時拒否又は撤回することができること。また、拒否・撤回によって被験者が不利な扱いを受けたり、治験に参加しない場合に受けるべき利益を失うことはないこと
(9) 治験への参加後、治験の参加を取りやめる場合の治験薬の取扱いに関する事項
(10) モニター、監査担当者、治験審査委員会等及び規制当局が医療に係る原資料を閲覧できること。その際、被験者の秘密は保全されること。また、同意文書に被験者が記名押印又は署名することによって閲覧を認めたことになること
(11) 治験の結果が公表される場合であっても、被験者の秘密は保全されること
(12) 被験者が治験及び被験者の権利に関してさらに情報の入手を希望する場合又は治験に関連する健康被害が生じた場合に照会すべき又は連絡をとるべき実施医療機関の相談窓口
(13) 治験に関連する健康被害が発生した場合に被験者が受けることのできる補償及び治療
(14) 当該治験の適否等について調査審議を行う治験審査委員会の種類、各治験審査委員会において調査審議を行う事項、その他当該治験に係る治験審査委員会に関する事項
(15) 治験に参加する予定の被験者数
(16) 治験への参加の継続について被験者の意思に影響を与える可能性のある情報が得られた場合には速やかに被験者に伝えること
(17) 治験への参加を中止させる場合の条件又は理由
(18) 被験者が費用負担をする必要がある場合にはその内容
(19) 被験者に金銭等が支払われる場合にはその内容（支払額算定の取決め等）
(20) 被験者が守るべき事項

4.3 同意取得の方法

(1) 治験責任（分担）医師は治験実施に先立ち、治験審査委員会にて承認を得た最新の同意説明文書を対象となる被験者及びその代諾者に手渡して、十分に説明する。また、治験協力者が補足的に説明することもできる。なお、説明に際し、本治験に関する説明文書に基づき、被験者及びその代諾者が理解できるように可能な限り平易な言葉を用いて説明し、被験者及びその代諾者の質問に対して十分に答えなければならない。被験者及びその代諾者が内容をよく理解したことを確認した上で、本治験への参加について被験者の自由意思による同意をその代諾者から文書にて得る。併せて、被験者が 7 歳以上においては、アセントを本人から文書にて得る。
(2) 同意文書には説明を行った治験責任（分担）医師及び被験者の代諾者が、記名押印又は署名し、各自日付を記載する。併せて、被験者が 7 歳以上においては、アセント文書には説明を行った治験責任（分担）医師及び被験者が署名し、各自日付を記

載する。いずれの場合も、治験協力者が補足的に説明を行った場合は、当該治験協力者も記名押印又は署名し、日付を記載する。

(3) 治験責任（分担）医師は、被験者の代諾者が説明文書を読むことができない場合、公正な立会人を立ち会わせた上で十分に説明し、被験者の自由意思による同意を得る。また、立会人も同意文書に記名押印又は署名し、日付を記載する。

(4) 治験責任（分担）医師は、被験者が治験に参加する前に、記名押印又は署名と日付が記載された同意文書の写しと説明文書を被験者の代諾者に交付する。併せて、被験者が7歳以上においては、署名と日付が記載されたアセント文書の写しと説明文書を被験者の代諾者に交付する。また、同意文書の原本は当該実施医療機関の規定に従って適切に保管する。

4.4 同意説明文書の改訂

(1) 被験者の同意に関連し得る新たな重要な情報が得られた場合に、治験責任医師は、速やかに当該情報に基づき同意説明文書改訂の要否を判断する。

(2) 治験責任医師は、同意説明文書を改訂する必要があると認める場合には、速やかに同意説明文書を改訂し、改めて治験審査委員会の承認を得なければならない。

(3) 上記 (2) に該当する場合において、治験責任（分担）医師は、既に治験に参加している被験者及びその代諾者に当該情報を口頭で速やかに伝え、治験に継続して参加するか否かについて確認し、診療録に記録する。

(4) 治験責任（分担）医師は、既に治験に参加している被験者及びその代諾者に対して、治験審査委員会にて改めて承認された同意説明文書を用いて説明し、治験への参加の継続について被験者からの自由意思による同意を文書にて得る。

(5) 初回の同意取得時と同様に、説明を行った治験責任（分担）医師、被験者又はその代諾者が記名押印又は署名し、各自日付を記載する。治験協力者が補足的に説明を行った場合は、当該治験協力者も記名押印又は署名し、日付を記載する。

(6) 治験責任（分担）医師は、被験者又はその代諾者が説明文書を読むことができない場合、公正な立会人を立ち会わせた上で十分に説明し、被験者の自由意思による同意を得る。また、立会人も同意文書に記名押印又は署名し、日付を記載する。

(7) 治験責任（分担）医師は、記名押印又は署名と日付が記載された同意文書の写しと説明文書を被験者及びその代諾者に交付する。また、同意文書の原本は当該実施医療機関の規定に従って適切に保管する。

5. 登録

5.1 登録の手順

以下の手続きに従って、登録を行う。

治験責任（分担）医師は、本治験の対象として適格となる可能性があると判断した被験者及びその代諾者から文書による同意を取得し、スクリーニング検査を実施した後、登録基準を満たした場合には、登録・適格確認に必要項目をすべて記録する。登録センターにおいて、治験責任（分担）医師の入力情報を基に適格性が判定される。記録内容に疑義がある場合、登録センターは直ちに、治験責任（分担）医師に確認を行う。

登録基準を満たした場合には、登録センターは、登録順に最初の 6 例は「治験薬 1 日 1 回 投与群」、その後の 6 例は「治験薬 1 日 2 回 投与群」とし、治験責任（分担）医師に連絡する。

登録基準を満たさなかった場合には、治験責任（分担）医師は、その理由を原資料に記録する。

5.2 登録に際しての注意事項

(1) 本治験は第 1 相試験であり、塩酸メクリジンの効果は期待できず、副作用などの不利益のみが生じる可能性があることを被験者及びその代諾者が理解できない場合、被験者の登録は許容できない。

(2) 登録に必要な項目の記入が不十分なときは、すべて満たされるまで登録は受け付けられない。

(3) 一度登録された被験者の登録の取り消しは行わない。

(4) 誤登録・重複登録が判明した場合には、速やかに登録センターに連絡をする。

(5) 重複登録がなされた場合は、先の登録を優先する。

5.3 本治験の被験者として不適格と判断された場合

治験責任（分担）医師は、被験者として不適格と判断した場合には、必要に応じて適切な治療を行う。同意取得後、登録に至らなかった被験者については、当該被験者の症例報告書を作成しない。

6. Method of trial

6.1 Type of clinical trial

6.1.1 Type of clinical trial

Phase 1 study

6.1.2 Trial design

Unblinded two group trial

【設定根拠】 本治験は、安全性の検討を行うとともに、軟骨無形成症の小児患者の薬物動態データを収集し、蓄積性の検討を行う。すなわち、1 日 1 回、及び 1 日 2 回投与のメクリジンの血漿中薬物濃度から、14 日間反復投与した場合の薬物動態のシミュレーションを実施し、反復投与試験開始のための、用量設定の根拠となるデータを取得するとともに、安全性データの充足につなげることが目的である。また、投与回数（1 日 1 回、又は 1 日 2 回）の検討を実施する予定であり、1 日 1 回投与と、1 日 2 回投与の 2 群を設定する。薬物濃度が指標であり盲検化は不要と判断した。

6.2 Medication methods

・Once a day group：Take meclizine once a day in the morning

Before administration of meclizine, fasting for 10 hours or more (without breakfast) and take a meclizine tablet with a fixed amount of water (approximate 150mL) without chewing, and fast until one hour after administration

After one our of administration, take a diet managed by the medical institution

However, there is no restriction of water intake throughout the day

・Twice a day group：Take meclizine twice a day, in the morning and in the evening

Take a regular diet managed by the medical institution one hour prior to administration of the study drug

Take a meclizine tablet with a fixed amount of water (approximate 150mL) without chewing, and fast until 4 ours after administration

After 4 ours of administration, take a regular diet managed by the medical institution

Take a regular diet managed by the medical institution before the second administration

Take a meclizine tablet with a fixed amount of water (approximate 150mL) without chewing

However, there is no restriction of water intake throughout the day

Record the time of accurate food intake and time of meclizine administration to the original data

6.3 evaluation items

6.3.1 Primary outcome

Pharmacokinetics：Plasma concentration of meclizine

① Cmax (during 24 hours)

② Tmax (during 24 hours)

③ t 1/2 (during 24 hours)

④ AUC (during 24 hours)

Accumulation effects（7 days after administration）

Safety ：Name, extent, and rate of adverse events or side effects

6.3.2 Secondary outcome

Simulation of plasma concentration

（Simulate pharmacokinetics with repeated administration for 14 days based on the plasma concentration of meclizine after once or twice a day administration）

7. Target number of cases and trial period

7.1 Target number of cases

12 cases（Once aday group : 6 cases、Twice a day group : 6 cases）

治験終了までの期間に脱落や中止症例が出た場合には症例数の追加を行う。また、25mg 錠 1 日 1 回投与群 6 例の評価期間が終了し、安全性を確認したのち 25mg 錠 1 日 2 回投与群の治験を実施する。12 例の評価期間が終了した場合には、あらかじめ同意を得ていても治験に参加できないことがある（同意取得から治験開始までの期間が長くなった場合などを想定する）。

【設定根拠】

第 1 相試験の薬物動態解析における標準的な例数として 1 群 6 例を設定した。

7.2 Trial period

From June, 2018 to March, 2019

8. 治験薬

8.1 治験薬の概要

治験薬の名称：MECLIZIN

一般名：メクリジン塩酸塩

和名：塩酸メクリジン　（局外規）

国際名称：Meclizine (Meclozine) HCl

分子式：C25H27Cl N2, 2Cl H

分子量：481.8

構造式：

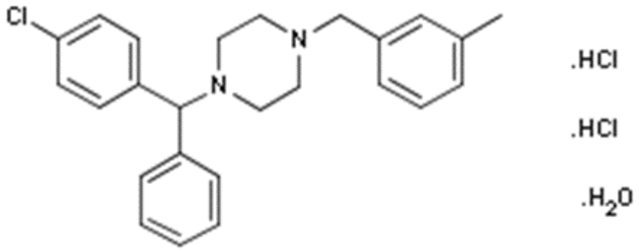

製剤：一般用医薬品（第二種医薬品）として承認されているソラシドンを使用する。

8.2 治験薬の製剤組成

ソラシドン（明治薬品）：

1 錠中に塩酸メクリジン 25mg を含有

（添加物）D-マンニトール、セルロース、カルメロース Na、硬化油、ステアリン酸 Mg、I-メントール

有効期限：室温で製造後 60 ヵ月（2022 年 12 月まで）

8.3　治験薬の包装及び表示

治験薬ラベルには治験用であること、識別記号、一般的名称、治験責任医師の氏名、所属、職名及び住所を表示する。

**治 験 用**	**軟骨無形成症患者（小児）に対する塩酸メクリジン製剤の安全性及び薬物動態の検討**<br>**本治験以外の目的で使用しないこと**
**識別記号**:	MECLIZIN
**製造番号**:	17040
**一般的名称**:	**メクリジン塩酸塩**
**保管及び取扱いの注意**:	直射日光・湿気を避けて保管
**有効期間**:	2022 年 10 月
**治験責任医師**:	**名古屋大学医学部附属病院　整形外科**<br>**准教授　鬼頭浩史**<br>**愛知県名古屋市昭和区鶴舞町** 65

8.4 治験薬の取扱い、保管及び管理

自ら治験を実施する者は、治験薬として一般用医薬品（第二種医薬品）として承認されているソラシドンの市販品を購入する。治験薬管理者は、治験責任医師が作成した「治験薬の管理に関する手順書」に従い、直射日光の当たらない湿気の少ない涼しい場所で治験薬を保管、管理する。

保管温度：室温

9.　併用薬・併用療法

9.1 併用を禁止・制限する薬剤・療法

治験薬投与開始 7 日前から評価期間終了までの期間、次の薬剤の併用を禁止とする。

① FGFR3 シグナルを抑制する成績が発表されている薬剤 （国内未承認の CNP アナログ製剤、スタチン製剤等）

② 塩酸メクリジンを含有する薬剤

治験薬投与開始 24 時間前から投与終了後 24 時間以内は、次の薬剤の併用を禁止とする。

③ かぜ薬，解熱鎮痛薬，鎮静薬，鎮咳去痰薬，抗ヒスタミン剤を含有する内服薬等（鼻炎用内服薬，アレルギー用薬等）

同意取得後から評価期間終了までの期間において、以下の治療を禁止する。

④ 同意取得時に成長ホルモン製剤による治療を受けている被験者は、評価期間終了までに治療の中止、変更を禁止する。

⑤ 骨延長術による治療は禁止する。

被験者の治療のため、これらの薬剤、療法を実施する場合は、治験を中止する。

【設定根拠】

①②FGFR3 シグナルの過剰抑制を防ぐために設定した。

②薬物動態の評価を適切に行うため設定した。

③ソラシドンの添付文書の記載に従った。

④有効性の評価は実施しないので、成長ホルモン剤の使用は禁止しない。有害事象の観察・評価を正確に行うため、治験期間中の治療の中止、変更は禁止した。

⑤有害事象の観察・評価を正確に行うため治験期間中は禁止した。

9.2 併用薬・併用療法の記録

治験責任（分担）医師又は治験協力者は、投与開始 7 日前から評価期間終了までの期間に併用した薬剤について、以下の内容を記録する。ただし、注射剤の溶解等を目的とした薬剤、補液、採血ルート確保のための薬剤及び消毒薬は記録不要とする。

併用薬：薬剤名（商品名）、投与期間、投与量、使用目的

なお、併用療法については、同意取得後から治験期間終了までのうち治療開始から最終評価の期間に併用した治療に関し、療法名、治療期間、治療目的について記録する。

10. 安全性評価委員会

有識者からなる安全性評価委員会を組織する。安全性評価委員には本治験の関係者は含まない。安全性評価委員会の評価手順については、別途作成する手順書に定める。

11. 投与・検査・観察

11.1 投与・検査・観察スケジュール

1 日 1 回投与群

項目	同意取得	スクリーニング期間		評価期間													
				1日目											2日目・24時間後	投与終了7日後	中止時
				朝投与													
		スクリーニング	登録	投与前	投与1時間後	投与2時間後	投与3時間後	投与4時間後	投与8時間後	投与10時間後	投与11時間後	投与12時間後	投与13時間後	投与14時間後			
【参考】朝9時投与の場合の時刻				9 AM	10 AM	11 AM	12 AM	1 PM	5 PM	7 PM	8 PM	9 PM	10 PM	11 PM	9 AM		
同意取得	●																
被験者背景調査		●															
既往歴、合併症		●															
登録			●														
治験薬服用				●													
身長		●															
体重		●		●											●	●	●
バイタルサイン		●		●	●	●	●	●	●	●					●	●	●
12誘導心電図		●		●*											●	●	●
胸部レントゲン 検査		●															
血液学的検査		○		●												●	●
血液生化学的検査		○		●												●	●
尿検査		○		●												●	●
薬物濃度測定				●	●	●	●	●	●	●					●	●	●
有害事象					●	●	●	●	●	●	●	●	●	●	●	●	●
併用薬・併用療法		●	●	●	●	●	●	●	●	●	●	●	●	●	●	●	●

○ :投与開始前28日以内に日常診療として検査している場合は、既存資料としての使用について被験者の同意が得られた場合、当該データをスクリーニング時のデータとして使用する。
* :スクリーニング検査を治験薬投与日から28日以内に実施している場合には、スクリーニング時のデータを採用する。

1 日 2 回投与群

項目	同意取得	スクリーニング期間		評価期間														中止時
				1日目												2日目・24時間後	投与終了7日後	
				朝投与							夕投与							
		スクリーニング	登録	投与前	投与1時間後	投与2時間後	投与3時間後	投与4時間後	投与8時間後	投与10時間後	投与前	投与1時間後	投与2時間後	投与3時間後	投与4時間後			
【参考】朝9時、夕7時投与の場合の時刻				9 AM	10 AM	11 AM	12 AM	1 PM	5 PM	7 PM		8 PM	9 PM	10 PM	11 PM	9 AM		
同意取得	●																	
被験者背景調査		●																
既往歴、合併症		●																
登録			●															
治験薬服用				●						●								
身長		●																
体重		●		●												●	●	●
バイタルサイン		●		●	●	●	●	●	●	●		●	●	●	●	●	●	●
12誘導心電図		●		●*												●	●	●
胸部レントゲン検査		●																
血液学的検査		○		●													●	●
血液生化学的検査		○		●													●	●
尿検査		○		●													●	●
薬物濃度測定				●	●	●	●	●	●	●		●	●	●	●	●	●	●
有害事象					●	●	●	●	●	●		●	●	●	●	●	●	●
併用薬・併用療法		●	●	●	●	●	●	●	●	●		●	●	●	●	●	●	●

○ :投与開始前28日以内に日常診療として検査している場合は、既存資料としての使用について被験者の同意が得られた場合、当該データをスクリーニング時のデータとして使用する。
* :スクリーニング検査を治験薬投与日から28日以内に実施している場合には、スクリーニング時のデータを採用する。

11.2 検査・観察項目及び時期

11.2.1 スクリーニング期間

同意取得後から投与開始 28 日以内以下の検査・観察を実施する。

(1) 被験者背景：性別、生年月日

(2) 身長

(3) 体重

(4) 既往歴、合併症

(5) バイタルサイン（血圧、脈拍数、体温）：仰臥位で測定する。

(6) 12 誘導心電図

(7) 胸部レントゲン撮影（正面）

(8) 血液学的検査：赤血球数、白血球数、血小板数、ヘモグロビン量、ヘマトクリット値

(9) 血液生化学検査： TP、Alb、BUN、Cre、CRP、Na、K、Cl、ALT、AST

(10) 尿検査：pH、蛋白、グルコース、ウロビリノーゲン、潜血

(11) 併用薬、併用療法の確認

「（5）血液学的検査」「（6）血液生化学検査」「（7）尿検査」については、投与開始前 28 日以内に日常診療として検査している場合は、既存資料としての使用について被験者の同意が得られた場合、当該データをスクリーニング時のデータとして使用する。
「(4) 既往歴、合併症」について、既往歴は同意取得の 1 年前から投与開始までに治癒した疾患、合併症は投与開始日に合併している疾患とする。

11.2.2 治験薬投与日（朝投与前）

(1) 体重

(2) バイタルサイン（血圧、脈拍数、体温）：仰臥位で測定する。

(3) 12 誘導心電図

(4) 血液学的検査：赤血球数、白血球数、血小板数、ヘモグロビン量、ヘマトクリット値

(5) 血液生化学検査： TP、Alb、BUN、Cre、CRP、Na、K、Cl、ALT、AST

(6) 尿検査：pH、蛋白、グルコース、ウロビリノーゲン、潜血

(7) 薬物濃度測定

(8) 併用薬、併用療法の確認

なお、薬物動態評価のための検体の採取、取扱い及び搬送手順については、別途手順書を参照することとする。また、「11.2.2 治験薬投与日（朝投与前)」～「11.2.14 治験薬投与終了 7 日後（評価期間終了時)」または「11.2.15 中止時」において、薬物動態測定の為の

採血時刻について原資料へ記録する。

11.2.3 治験薬投与日（朝投与 1 時間後）

(1) バイタルサイン（血圧、脈拍数、体温）: 仰臥位で測定する。
(2) 薬物濃度測定
(3) 有害事象
(4) 併用薬、併用療法の確認

11.2.4 治験薬投与日（朝投与 2 時間後）

(1) バイタルサイン（血圧、脈拍数、体温）: 仰臥位で測定する。
(2) 薬物濃度測定
(3) 有害事象
(4) 併用薬、併用療法の確認

11.2.5 治験薬投与日（朝投与 3 時間後）

(1) バイタルサイン（血圧、脈拍数、体温）: 仰臥位で測定する。
(2) 薬物濃度測定
(3) 有害事象
(4) 併用薬、併用療法の確認

11.2.6 治験薬投与日（朝投与 4 時間後）

(1) バイタルサイン（血圧、脈拍数、体温）: 仰臥位で測定する。
(2) 薬物濃度測定
(3) 有害事象
(4) 併用薬、併用療法の確認

11.2.7 治験薬投与日（朝投与 8 時間後）

(1) バイタルサイン（血圧、脈拍数、体温）: 仰臥位で測定する。
(2) 薬物濃度測定
(3) 有害事象
(4) 併用薬、併用療法の確認

11.2.8 治験薬投与日（朝投与 10 時間後/夕投与前）

(1) バイタルサイン（血圧、脈拍数、体温）: 仰臥位で測定する。
(2) 薬物濃度測定
(3) 有害事象
(4) 併用薬、併用療法の確認

11.2.9 治験薬投与日（朝投与 11 時間後/夕投与 1 時間後）
・1 日 2 回投与群においては以下の検査・確認を実施する
(1) バイタルサイン（血圧、脈拍数、体温）：仰臥位で測定する。
(2) 薬物濃度測定
(3) 有害事象
(4) 併用薬、併用療法の確認
・1 日 1 回投与群においては(3)(4)の確認を実施する

11.2.10 治験薬投与日（朝投与 12 時間後/夕投与 2 時間後）
・1 日 2 回投与群においては以下の検査・確認を実施する
(1) バイタルサイン（血圧、脈拍数、体温）：仰臥位で測定する。
(2) 薬物濃度測定
(3) 有害事象
(4) 併用薬、併用療法の確認
・1 日 1 回投与群においては(3)(4)の確認を実施する

11.2.11 治験薬投与日（朝投与 13 時間後/夕投与 3 時間後）
・1 日 2 回投与群においては以下の検査・確認を実施する
(1) バイタルサイン（血圧、脈拍数、体温）：仰臥位で測定する。
(2) 薬物濃度測定
(3) 有害事象
(4) 併用薬、併用療法の確認
・1 日 1 回投与群においては(3)(4)の確認を実施する

11.2.12 治験薬投与日（朝投与 14 時間後/夕投与 4 時間後）
・1 日 2 回投与群においては以下の検査・確認を実施する
(1) バイタルサイン（血圧、脈拍数、体温）：仰臥位で測定する。
(2) 薬物濃度測定
(3) 有害事象
(4) 併用薬、併用療法の確認
・1 日 1 回投与群においては(3)(4)の確認を実施する

11.2.13 治験薬投与 2 日目（朝投与 24 時間後）
(1) 体重
(2) バイタルサイン（血圧、脈拍数、体温）：仰臥位で測定する。

(3) 12 誘導心電図
(4) 薬物濃度測定
(5) 有害事象
(6) 併用薬、併用療法の確認

11.2.14 治験薬投与終了 7 日後（評価期間終了時）
(1) 体重
(2) バイタルサイン（血圧、脈拍数、体温）：仰臥位で測定する。
(3) 12 誘導心電図
(4) 血液学的検査：赤血球数、白血球数、血小板数、ヘモグロビン量、ヘマトクリット値
(5) 血液生化学検査： TP、Alb、BUN、Cre、CRP、Na、K、Cl、ALT、AST
(6) 尿検査：pH、蛋白、グルコース、ウロビリノーゲン、潜血
(7) 薬物濃度測定
(8) 有害事象
(9) 併用薬、併用療法の確認

11.2.15 中止時
中止時の扱い：「14.1 被験者の中止基準」で述べるような状況の場合、治験を中止する。治験薬投与後に中止する際には、下記の中止時検査を行う。
(1) 体重
(2) バイタルサイン（血圧、脈拍数、体温）：仰臥位で測定する。
(3) 12 誘導心電図
(4) 血液学的検査：赤血球数、白血球数、血小板数、ヘモグロビン量、ヘマトクリット値
(5) 血液生化学検査： TP、Alb、BUN、Cre、CRP、Na、K、Cl、ALT、AST
(6) 尿検査：pH、蛋白、グルコース、ウロビリノーゲン、潜血
(7) 薬物濃度測定
(8) 有害事象
(9) 併用薬、併用療法の確認

12. 有害事象

12.1 有害事象等の定義

有害事象とは、治験薬を投与した被験者に生じたすべての疾病若しくは障害又はこれらの兆候であり（臨床検査値の異常変動を含む）、治験薬との因果関係の有無を問わない。本治

験では、治験薬の投与開始から評価期間終了／中止時までに発現したあらゆる好ましくない医療上のできごとを有害事象として取り扱う。

12.2 有害事象の評価及び基準

12.2.1 発現日

症状が認められた日又は臨床検査値異常変動等が認められた検査日とする。

12.2.2 程度

有害事象の程度は、次の基準で分類する。

1. 軽　度：被験者の日常生活に影響がない程度
2. 中等度：被験者の日常生活にその事象が原因で多少の支障がある程度
3. 高　度：被験者の日常生活がその事象が原因でできない程度

12.2.3 重篤性

1. 非重篤
2. 重篤

重篤な有害事象は、次のいずれかに該当する有害事象とする。

1. 死亡
2. 死亡につながるおそれのあるもの
3. 治療のために病院又は診療所への入院又は入院期間の延長が必要となるもの
4. 障害
5. 障害につながるおそれのあるもの
6. 1 から 5 に掲げる症例に準じて重篤であるもの
7. 後世代における先天性の疾病又は異常

ただし、治験開始前より予定された入院又は入院期間の延長が必要となるもの、また有害事象の治療を目的としない入院又は入院期間の延長は除く。

12.2.4 処置の有無

1. 処置あり
2. 処置なし

12.2.5 処置内容

1. 外科的処置
2. 薬物治療
3. その他

12.2.6 治験薬との因果関係

治験責任（分担）医師は、治験薬に当該有害事象を引き起こす「合理的な可能性」が存在するか否かを評価する。評価は、原疾患、合併症等の基礎疾患の自然経過、併用療法、その他の危険因子等、治験薬以外の原因及び治験薬使用と事象発現との時間的関連を勘案し、次の様に分類する。

1. 関連あり
2. 関連なし
3. 不明

12.2.7 転帰

有害事象の転帰は、次の 6 段階で分類する。

1. 回復
2. 軽快
3. 未回復
4. 回復したが後遺症あり
5. 死亡
6. 不明

12.2.8 転帰日

転帰日は、転帰に応じ、それぞれ次の基準で判断する。

回　復：回復した日。ただし、回復した日が特定できない場合は回復を確認又は判断した日

軽　快：軽快を確認又は判断した日

未回復：未回復を確認又は判断した日

後遺症あり：後遺症ありを確認又は判断した日

死　亡：死亡日。ただし、死亡日が特定できない場合は死亡を確認又は判断した日

不　明：当該有害事象以外の原因で被験者死亡のため転帰が不明だった場合は死亡日。その他の場合は確認又は判断した日

12.3 有害事象の追跡

有害事象は、事象が消失するか、治験責任（分担）医師が追跡の必要がないと判断するまで追跡する。治験薬との因果関係があるが追跡不要と判断した場合は、その理由を記録する。

12.4 有害事象の記録事項

有害事象を認めた場合、治験責任（分担）医師は、その有害事象名※、発現日、程度、重篤性、重篤と判断された場合はその理由、転帰、転帰日、治験薬との因果関係、処置等を行ったものについてはその内容（薬剤名、療法等）を記録する。また、有害事象の転帰が回復又は回復したが後遺症あり、死亡以外で追跡調査不要と判断した場合、その理由を記録する。

※「有害事象名」は以下の基準に従う。

- ✓ 原則として診断名を用いる。
- ✓ 診断名が明確でない場合は症状名を用いる。
- ✓ 複数の症状が発現していて、それらが1つの診断名で示すことができる場合は、その診断名を用いる。
- ✓ 外科的処置等は有害事象とせず、外科的処置等を必要とする病気や症状が確認されている場合は、それを有害事象とする。

13. 被験者の安全性の確保

13.1 予想される有害事象

本治験では、治験薬に関連する有害事象として、当該事象等の発生、あるいは発生数、発生頻度、発生条件等の発生傾向が本治験薬概要書あるいは下記から予測できないものを「未知」、予測できるものを「既知」とする。ただし、厚生労働大臣へ報告した安全性情報を治験薬概要書の別冊として取扱い、当該報告文書の作成日をもって当該事象を「既知」と取扱う。

13.1.1 治験薬に関連する有害事象

治験薬として用いるソラシドンの添付文書に記載がある有害事象の症状は以下のとおりである。

皮膚：発疹・発赤, かゆみ
泌尿器： 排尿困難
その他：口のかわき, 眠気

また、ソラシドンと同一有効成分の一般用医薬品センパアＳ（大正製薬）の添付文書に記載がある有害事象の症状は以下のとおりである。

皮膚：発疹・発赤，かゆみ
精神神経系：頭痛
泌尿器：排尿困難
その他：顔のほてり、異常なまぶしさ、口のかわき、便秘、眠気、目のかすみ

これらの症状の出現、並びにこれらの症状を伴う疾患については「既知」とする。

13.2 重篤な有害事象発現時の対応

治験中に重篤な有害事象が発現した場合は、治験責任（分担）医師は、被験者に対して直ちに適切な処置を行う。

① 重篤な有害事象の発生を認めたとき又はその発生のおそれがあると認めた場合、治験責任医師は直ちに実施医療機関の長に報告する。また、治験分担医師、治験調整事務局にも、その発生経緯及び被験者等について情報を提供する。

② 治験責任医師は、当該有害事象に関する追加情報が得られた場合には、重篤な有害事象に関する報告書により可能な限り速やかに実施医療機関の長に追加報告を行うとともに治験分担医師、治験調整事務局に情報を提供する。

③ 実施医療機関の長は治験責任医師から報告を受けたときは、治験の継続について治験審査委員会の意見に基づき速やかに治験責任医師に対して対応を指示する。

④ 治験責任医師は、その発生経緯及び被験者等について、医薬品、医療機器等の品質、有効性及び安全性の確保等に関する法律施行規則第 273 条の 2 第 1 項に規定される報告対象に該当すると判断した場合は、「安全性情報の取扱いに関する手順書」に従い、厚生労働大臣に報告をする。

13.3 被験者の他の主治医への連絡等

治験責任（分担）医師は、被験者がこの治験以外に医師の診察を受けているか否かを確認する。他の医師に診察を受けている場合には、被験者の同意の下に、当該医師に被験者の治験参加について連絡する。

14. 被験者の中止基準及びその手順

14.1 被験者の中止基準

以下の中止基準に該当する場合は、治験を中止する。

(1)被験者又は代諾者の自由意思による同意撤回の申し入れがあった場合

(2)治験責任（分担）医師により、原疾患あるいは合併症の悪化又は重篤な有害事象等の発現のため治験の継続が困難と判断された場合

(3)GCP 違反、選択基準違反又は除外基準抵触等、治験実施計画書からの重大な逸脱が判明した場合

(4)治験実施計画書の遵守が不可能になった場合

(5)治験期間中に薬物動態・蓄積性又は安全性の評価の上で、対象として不適切であることが判明した場合

(6)その他、治験責任（分担）医師により治験の継続が困難と判断された場合

【設定根拠】

(1)～(6)　治験を倫理的に実施するため設定した。

14.2 中止の手順

治験責任（分担）医師は、治験を中止した場合には、当該被験者に対し適切な措置を講ずる。

治験期間中に中止した場合には中止時検査を実施する。

治験中止後も持続する治験薬との因果関係が否定出来ない有害事象については、事象が回復するか、治験責任（分担）医師が追跡の必要がないと判断するまで、追跡する。

治験責任（分担）医師は、中止日及び中止理由を記録する。なお、中止日とは、中止時の評価を実施した日（評価日）とするが、中止時評価が行えなかった場合は中止と判断した日とする。

中止時の規定の検査・観察ができなかった被験者又は中止後に来院のない被験者については、中止時検査未実施の理由及び中止後の経過を、文書（封書）又は電話等により追跡調査し、その内容を記録する。

15. 解析

15.1 対象集団

15.1.1　薬物動態の解析対象集団

(1) 最大の解析対象集団 (full analysis set：FAS)

本治験における主たる解析対象集団は、本治験に登録された被験者のうち次のいずれかに該当する被験者を除いた最大の解析対象集団 (full analysis set：FAS) とする。

- 重大な GCP 違反のある被験者
- 治験薬を一度も投与されなかった被験者
- 治験薬投与日（朝投与前）の薬物濃度の測定がない被験者
- 治験薬投与日（朝投与後）又は 治験薬投与翌日（朝投与 24 時間後）の時点の、薬物濃度の測定が全くない被験者

(2) 解析対象集団 (per protocol set：PPS)

FAS から次に該当する被験者を除いた解析対象集団 (per protocol set：PPS) を対象とする解析は、FAS を対象とする解析の結果の頑健性を確認するために実施する。

- 選択基準に該当しない又は除外基準に抵触する被験者
- 治験薬投与日（朝投与後）の薬物濃度の測定に欠損値がある被験者

15.1.2　薬物蓄積性の解析対象集団

(1)　最大の解析対象集団 (full analysis set：FAS)

本治験における主たる解析対象集団は、本治験に登録された被験者のうち次に該当する被験者を除いた最大の解析対象集団 (full analysis set：FAS) とする。

- 重大な GCP 違反のある被験者
- 治験薬を一度も投与されなかった被験者
- 治験薬投与日（朝投与前）の薬物濃度の測定がない被験者
- 治験薬投与翌日（朝投与 24 時間後）の薬物濃度の測定がない被験者

(2)　解析対象集団 (per protocol set：PPS)

FAS から次に該当する被験者を除いた解析対象集団 (per protocol set：PPS) を対象とする解析は、FAS を対象とする解析の結果の頑健性を確認するために実施する。

- 選択基準に該当しない又は除外基準に抵触する被験者

15.1.3　安全性の解析対象集団

安全性の解析は安全性解析対象集団を対象として行う。

安全性の解析対象集団は、治験薬が一度でも投与された被験者集団とする。

15.2 解析計画

本治験における主な解析事項は、以下の通りである。解析計画の詳細、変更及びデータの取り扱いの詳細を含む解析計画書をデータ固定前に作成する。

15.2.1 薬物動態評価項目

【主要評価項目】

① Cmax (投与 24 時間)

② Tmax (投与 24 時間)

③ t 1/2 (投与 24 時間)

④ AUC (投与 24 時間)

【副次的評価項目】

薬物動態のシミュレーション

（1 日 1 回、及び 1 日 2 回投与のメクリジンの血漿中薬物濃度から、14 日間反復投与した場合の薬物動態をシミュレーションする）

15.2.2 蓄積性評価項目

【主要評価項目】

薬物蓄積性（投与 7 日後）

15.2.3 安全性評価項目

有害事象の発生

安全性解析対象集団に対して、すべての有害事象の発現割合及び 95%信頼区間を算出する。信頼区間の計算には Clopper-Pearson 法を用いる。重篤な有害事象は被験者別に一覧表に示し、器官別大分類、基本語別に発現割合を算出する。

15.3 統計解析計画書の変更

統計解析計画をデータ固定前に変更する場合は、その変更理由を統計解析計画書及び総括報告書に記述する。データ固定後に解析方法の変更又は追加解析を実施する場合は、その理由を解析報告書及び総括報告書に記述し、計画されていた解析結果と区別する。

15.4 データの取り扱い

(1) 欠測値 欠測値については、データの補完を行わない。

(2) 薬物濃度測定用採血時期のずれの取り扱い

1 日 1 回投与群

治験薬投与日（朝投与前）：治験薬投与前 60 分のずれを許容する。
治験薬投与日（投与 1 時間後）：前後 10 分のずれを許容する。
治験薬投与日（投与 2 時間後）：前後 10 分のずれを許容する。
治験薬投与日（投与 3 時間後）：前後 10 分のずれを許容する。
治験薬投与日（投与 4 時間後）：前後 10 分のずれを許容する。
治験薬投与日（投与 8 時間後）：前後 30 分のずれを許容する。
治験薬投与日（投与 10 時間後）：前後 30 分のずれを許容する。
治験薬投与 2 日目（投与 24 時間後）： 前後 30 分のずれを許容する。
治験薬投与終了 1 週間後：前後 1 日のずれを許容する。

1 日 2 回投与群

治験薬投与日（朝投与前）：治験薬投与前 60 分のずれを許容する。
治験薬投与日（朝投与 1 時間後）：前後 10 分のずれを許容する。
治験薬投与日（朝投与 2 時間後）：前後 10 分のずれを許容する。
治験薬投与日（朝投与 3 時間後）：前後 10 分のずれを許容する。
治験薬投与日（朝投与 4 時間後）：前後 10 分のずれを許容する。

治験薬投与日（朝投与 8 時間後）：前後 30 分のずれを許容する。
治験薬投与日（夕投与前/朝投与 10 時間後）：夕投与直前の 30 分以内であれば、ずれを許容する。
治験薬投与日（夕投与 1 時間後）：前後 10 分のずれを許容する。
治験薬投与日（夕投与 2 時間後）：前後 10 分のずれを許容する。
治験薬投与日（夕投与 3 時間後）：前後 10 分のずれを許容する。
治験薬投与日（夕投与 4 時間後）：前後 10 分のずれを許容する。
治験薬投与 2 日目（朝投与 24 時間後）： 前後 30 分のずれを許容する。
治験薬投与終了 1 週間後：前後 1 日のずれを許容する。

16. 治験実施計画書の遵守、逸脱及び改訂

16.1 治験実施計画書の遵守

治験責任（分担）医師は、本治験実施計画書を遵守して治験を実施する。

16.2 治験実施計画書からの逸脱

治験責任（分担）医師は、治験審査委員会の事前の審査に基づく文書による承認を得ることなく、治験実施計画書からの逸脱を行ってはならない。ただし、治験責任（分担）医師は、被験者の緊急の危険を回避するため等医療上やむを得ない事情のために、治験審査委員会の事前の承認なしに治験実施計画書からの逸脱を行うことができる。

その際には、治験責任（分担）医師は、逸脱の内容及び理由を可能な限り早急に実施医療機関の長及び治験審査委員会に提出してその承認を得るとともに、実施医療機関の長の了承を文書で得なければならない。

治験責任（分担）医師は、治験実施計画書から逸脱した行為をすべて記録しなければならない。治験責任（分担）医師は、逸脱した行為のうち被験者の緊急の危険を回避するため、その他医療上やむを得ない理由により治験実施計画書に従わなかったものについてのみその理由を記載した文書を作成し、直ちに実施医療機関の長に提出し、その写しを保存する。

16.3 治験実施計画書の改訂

自ら治験を実施する者は、治験の進行中に治験実施計画書の変更が必要と認めた場合、治験実施計画書を改訂する。自ら治験を実施する者は、改訂内容について、実施医療機関の長に速やかに文書で通知し、実施医療機関の長を経由して治験審査委員会の承認を書面にて取得する。

治験審査委員会の意見に基づき実施医療機関の長から修正を指示された場合、自ら治験を実施する者は変更が妥当か否かを判断し、必要な場合は治験実施計画書を改訂する。改訂内容について、実施医療機関の長に速やかに文書で通知し、実施医療機関の長を経由して治

験審査委員会の承認を書面にて取得する。

17. 治験の中止又は中断

(1)　治験の中止又は中断の基準

治験責任医師は次の事例があった場合、実施医療機関全体又は一部の実施医療機関における治験実施継続の可否を検討する。

① 治験薬の品質及び安全性に関する事項、その他、治験を適正に行うために重要な情報を知った場合

② 治験実施計画書の変更が必要となり、実施医療機関がこれに対応できない場合

③ 治験審査委員会の意見に基づく実施医療機関の長からの治験実施計画書等に対する修正の指示があり、治験責任医師がこれを承諾できない場合

④ 治験審査委員会の判断に基づき、実施医療機関の長が治験の中止を指示した場合

⑤ 実施医療機関が GCP 又は本治験実施計画書に重大な又は継続的な違反を行った場合

(2)　治験責任医師による治験全体の中止又は中断

治験責任医師は、治験全体の中止又は中断を決定した場合、速やかにその旨及びその理由を規制当局に文書で通知する。実施医療機関の長は、治験責任医師より治験を中止又は中断する旨の通知を受けた場合、速やかにその旨及びその理由の詳細を治験審査委員会に文書で通知する。

治験責任医師は、治験が中止又は中断された場合、被験者に速やかにその旨を通知し、適切な治療を保証する。

治験を中止した場合の被験者への対応については、「14.2　中止の手順」に従う。

(3)　治験審査委員会による当該実施医療機関の治験の中止又は中断

治験審査委員会の判断により治験の中止又は中断を決定した場合には、速やかにその旨及びその理由の詳細を実施医療機関の長に文書で通知する。実施医療機関の長は、治験責任医師に速やかにその旨を文書で通知する。

18. CRF に関する事項

治験責任（分担）医師又は治験協力者は、別途提供される「症例報告書の入力、変更又は修正に関する手引き」に従って症例報告書を作成する。

症例報告書の提出時期

治験責任（分担）医師は、速やかに症例報告書を作成し、原本をデータマネジメント担当者に提出し、その写しを保管する。

19. 原資料等の直接閲覧

実施医療機関の長は、治験責任医師が実施させるモニタリング及び監査並びに治験審査委員会及び規制当局による調査を受け入れ、その際に、治験に関するすべての資料を直接閲覧に供することを保証する+。

20. 治験の品質管理及び品質保証

治験責任医師は、本治験の品質及び信頼性維持のために、別途作成する手順書に基づく「治験の品質管理」及び「治験の品質保証」を行わなければならない。また、実施医療機関は、治験責任医師による治験の品質管理及び品質保証に協力しなければならない。

治験の品質管理においては、モニタリング担当者は、「モニタリングの実施に関する手順書」に従い、本治験が実施医療機関の治験に係わる業務に関する手順書、最新の治験実施計画書及び GCP を遵守して実施されていることを確認する。

また、治験責任（分担）医師から報告された CRF の内容が正確かつ完全であることを、原資料等の治験関連記録に照らして検証できることを確認する。

また、治験が治験実施計画書及び GCP を遵守して行われていることを保証するため、監査担当者は手順書に従って監査を行い、品質管理が適切に行われていることを確認する。

21. 倫理

21.1 治験の倫理的実施

本治験はヘルシンキ宣言、医薬品医療機器等法、同施行規則、GCP、治験実施計画書を遵守して実施しなければならない。

21.2 治験審査委員会

治験審査委員会は、治験実施計画書、治験薬概要書、同意説明文書等の記載内容に基づき、倫理的、科学的及び医学的妥当性の観点から治験の実施及び継続について審査を行う。

21.3 被験者の秘密保全

被験者の登録及び CRF における被験者の特定は、被験者識別コードで行うとともに、治験の実施に係わる原資料の直接閲覧、医学雑誌への発表、規制当局等への資料提出等において、本治験に係わる者は被験者の秘密を保全する。

22. 記録等の保存

22.1 実施医療機関において保存する記録類

実施医療機関の長が定めた記録保管責任者は、実施医療機関において保存すべき治験に係る文書又は記録を次の 1) 又は 2) のうち遅い日までの期間保存する。ただし、治験責任医師がこれよりも長期間の保存を必要とする場合には、実施医療機関は、保存期間及び保存方法について治験責任医師と協議する。

1) 当該治験薬に係る製造販売承認日（開発の中止もしくは治験の成績が承認申請書に添付されない場合には開発中止が決定された、もしくは申請書に添付されない決定を行った日から 3 年が経過した日）
2) 治験の中止又は終了後 3 年が経過した日

なお、治験責任医師並びに治験実施医療機関が、塩酸メクリジン製剤の製造販売承認の取得を希望する企業に本治験の成績を、製造販売承認のために承認申請書に添付することを許諾した場合、上記保管期限終了後の保管については当該企業と話し合う。また、当該企業は本治験の成績を使用して製造販売承認が得られた場合、あるいは承認に至らず開発中止を決定した場合には、その旨を実施医療機関の長に文書で報告する。

22.2 治験責任医師において保存する記録類

治験責任医師は、治験責任医師において保存すべき治験に係る文書又は記録を次の 1) 又は 2) のうち遅い日までの期間保存する。

1) 当該治験薬に係る製造販売承認日（開発の中止もしくは治験の成績が承認申請書に添付されない場合には開発中止が決定された、もしくは申請書に添付されない決定を行った日から 3 年が経過した日）
2) 治験の中止又は終了後 3 年が経過した日

なお、治験責任医師並びに治験実施医療機関が、塩酸メクリジン製剤の製造販売承認の取得を希望する企業に本治験の成績を、製造販売承認のために承認申請書に添付することを許諾した場合、上記保管期限終了後の保管については当該企業と話し合う。また、当該企業は本治験の成績を使用して製造販売承認が得られた場合、あるいは承認に至らず開発中止を決定した場合には、その旨を実施医療機関の長に文書で報告する。

23. 治験の資金源と利益相反の管理

本治験実施に関し、治験責任（分担）医師は、治験薬の製造会社、薬物濃度測定機関からの資金提供、労務提供を受けていない。

なお、治験責任医師並びに治験実施医療機関は、塩酸メクリジン製剤の製造販売承認の取得を適正かつ迅速に推進する目的で、製造販売承認の取得を希望する企業に対し、本治験の成果を、製造販売承認のための承認申請書に添付することを許諾する場合がある。
今後、治験の実施及び成果に関して利益相反が生じる可能性に関して、実施医療機関の利益相反マネジメントポリシーに基づき、適切にこれを管理する。

24. 金銭の支払い

本治験において同意取得後に実施した診療報酬上評価されていない手術及び処置に関わる費用以外は保険診療の対象となる。

本治験の各検査項目に関わる費用、並びに軟骨無形成症治療のための入院及び手術に関わる費用は原則として被験者負担（保険診療）で行うが、保険診療に該当しない治験薬投与にかかる費用、本治験の薬物濃度測定に関わる費用について被験者負担は発生しない。被験者に対する負担軽減費の支払いについては、実施医療機関の規定に従う。

なお、本治験で使用する治験薬は、自ら治験を実施する者が、一般用医薬品（第二種医薬品）として承認されているソラシドンの市販品を購入して使用する。

25. 健康被害に対する補償

被験者に健康被害が生じた場合、実施医療機関はその治療に関する医療の提供等、必要かつ適切な処置を行う。その際、当該健康被害が治験薬との因果関係を否定できないと治験責任医師が判断した場合、治験責任医師はその補償費用を負担する。ただし、医療費、医療手当は支払われない。なお、健康被害が被験者の故意又は重大な過失によるものと判断した場合には、補償の対象外となる場合がある。

本治験に起因する健康被害による賠償責任が生じた場合の履行措置として、治験責任医師は、治験責任医師、治験分担医師及び実施医療機関が被保険者となる保険に加入する。また、治験責任（分担）医師は医師賠償責任保険に加入し、実施医療機関は病院賠償責任保険等に加入する。

26. 治験成果の帰属と結果の公表

本治験で得られるデータ等の情報は、治験責任（分担）医師の事前の了解を得ることなく、その一部又は全部を、その方法を問わず公表することはできない。

27. 治験の登録と進捗状況の報告

治験の実施に先立ち、事前に UMIN 臨床登録システムに本治験の主要なデザインを公開し、臨床試験登録する。

治験責任医師は、治験の進捗状況等を 1 年に 1 回、実施医療機関の治験審査委員会に報告する。また、治験を終了したときは、実施医療機関の長に終了の旨と結果の概要を文書により報告する。

28. 実施体制

別紙参照
