# Supplementary figures and images for: Pharmacokinetics and safety after once and twice a day doses of meclizine hydrochloride administered to children with achondroplasia
Source: PLoS One. 2020 Apr 13;15(4):e0229639. doi: 10.1371/journal.pone.0229639 (PMC7153885; doi:10.1371/journal.pone.0229639)

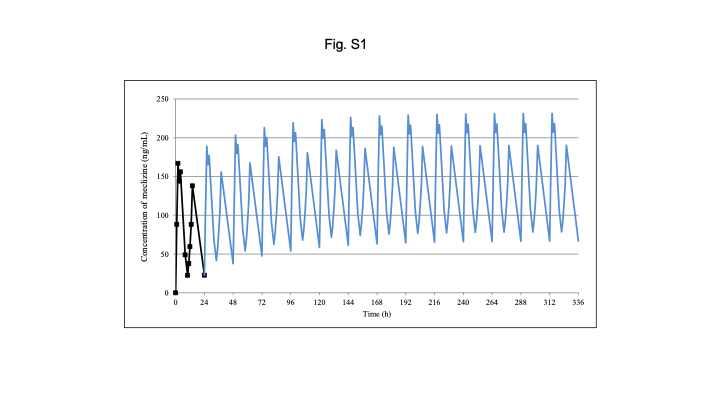

Supplement: S1 Fig — Plasma concentration apparently reached steady state around 10 days after the first dose. (TIFF) [file pone.0229639.s003.tiff]

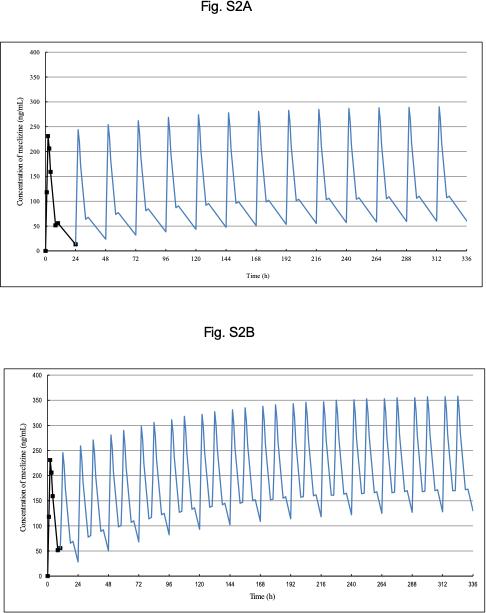

Supplement: S2 Fig — Simulated plasma concentration profile of meclizine at once a day (A) and twice a day (B) for 14 days multiple administrations using the plasma concentration of MEC-01 after single administration of meclizine hydrochloride 25 mg tablet. Plasma concentration reached steady state around 10 days and 12 days after the first dose at once a day and twice a day multiple administrations, respectively. (TIF) [file pone.0229639.s004.tif]

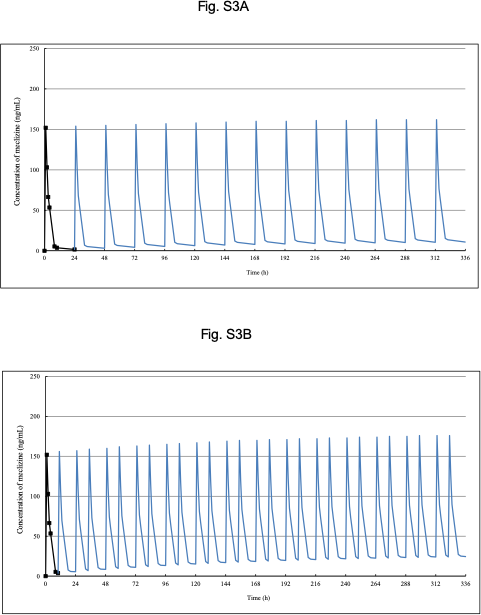

Supplement: S3 Fig — Simulated plasma concentration profile of meclizine at once a day (A) and twice a day (B) for 14 days multiple administrations using the plasma concentration of MEC-02 after single administration of meclizine hydrochloride 25 mg tablet. Plasma concentration reached steady state around 10 days after the first dose both at once and twice a day multiple administrations. (TIF) [file pone.0229639.s005.tif]

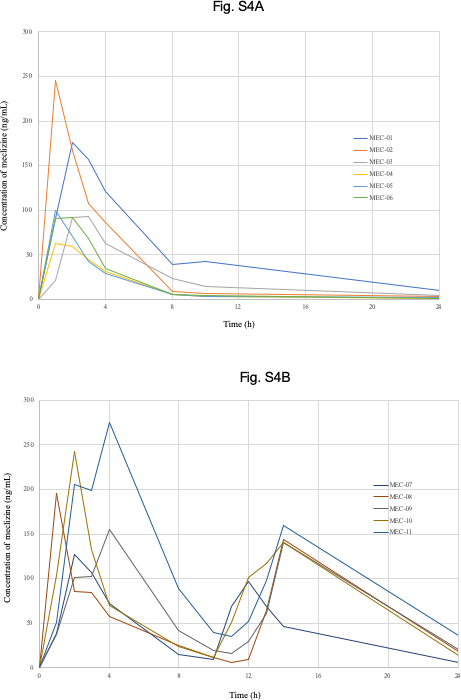

Supplement: S4 Fig — Simulated plasma concentration profile of meclizine after the 14th day administration using the elimination rate constant between 10 and 24 hours in “once a day group” (A) and 14 and 24 hours in “twice a day group” (B). (TIF) [file pone.0229639.s006.tif]

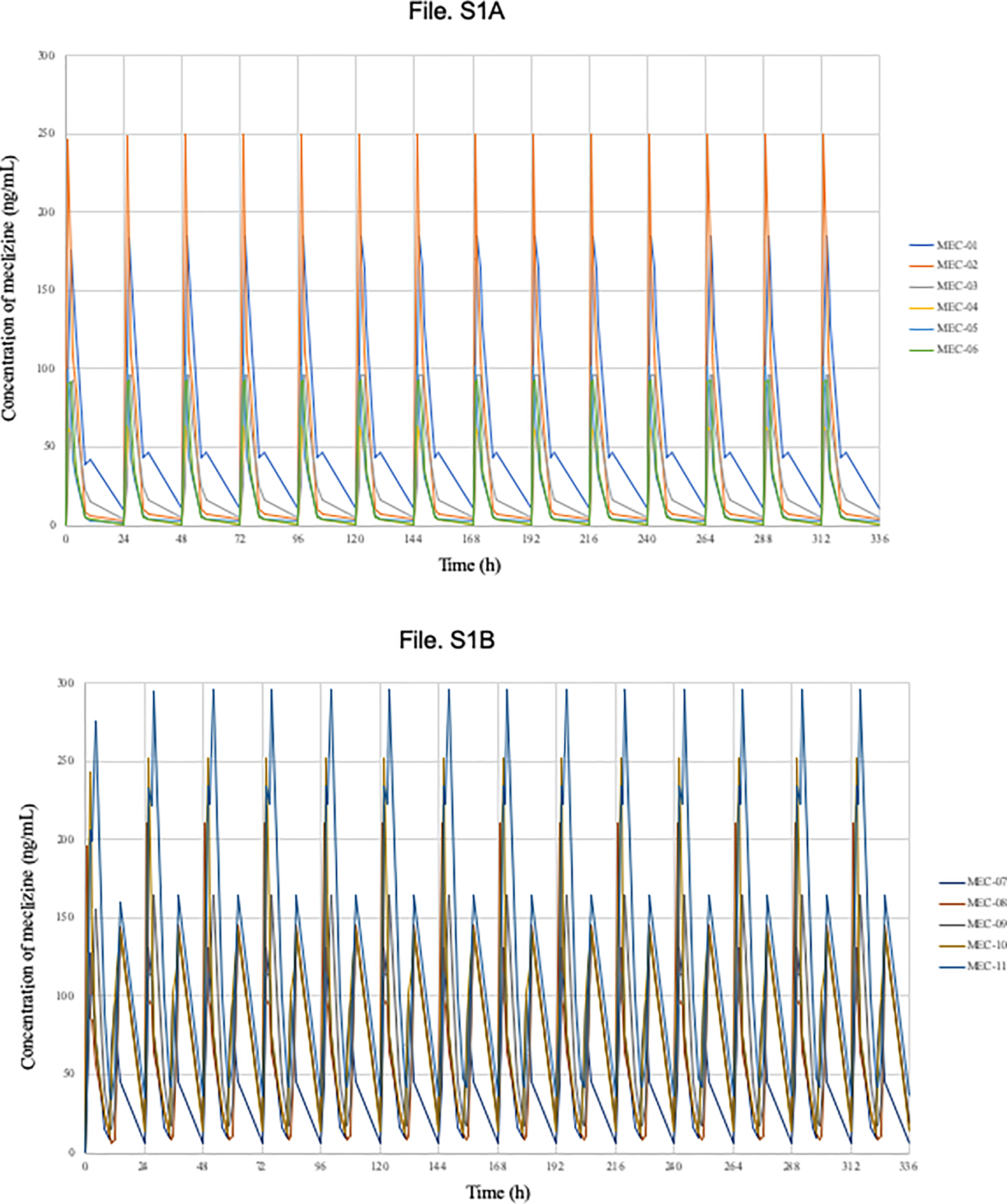

Supplement: S1 File — Simulated plasma concentration profile of meclizine at once a day (A) and twice a day (B) for 14 days multiple administrations in each individual. (TIF) [file pone.0229639.s008.tif]
