# Supplementary material for: Pharmacokinetics and safety after once and twice a day doses of meclizine hydrochloride administered to children with achondroplasia
Source: PLoS One. 2020 Apr 13;15(4):e0229639. doi: 10.1371/journal.pone.0229639 (PMC7153885; doi:10.1371/journal.pone.0229639)
Supplement: S1 Table — (PDF) [file pone.0229639.s007.pdf]

Subject	C_{max}	C_{min}	$AUC_{312-336h}$
MEC-01	185	11.1	1359
MEC-02	250	3.7	870
MEC-03	96	5.3	630
MEC-04	63	1.2	315
MEC-05	101	2.6	362
MEC-06	93	1	396
Mean	131.3	4.1	656
SD	71.1	3.8	403
CV%	54%	91%	61%
MEC-07	131	6.3	1058
MEC-08	211	18.2	1651
MEC-09	164	20.2	1865
MEC-10	252	14.0	1923
MEC-11	296	36.7	2957
Mean	210.8	19.1	1891
SD	66.2	11.2	687
CV%	31%	59%	36%

SD: standard deviation

CV: coefficient of variation
