# Supplementary material for: Pharmacokinetics and safety after once and twice a day doses of meclizine hydrochloride administered to children with achondroplasia
Source: PLoS One. 2020 Apr 13;15(4):e0229639. doi: 10.1371/journal.pone.0229639 (PMC7153885; doi:10.1371/journal.pone.0229639)
Supplement: Attachment — Submitted filename: Response to Reviewers.docx [file pone.0229639.s009.docx]

- Followings are point-by-point responses to the comments of the reviewers.
- The sentences and words we changed are written in red in the revised manuscript.
- Line numbers noted below refer to the revised manuscript.
- We changed Figures 2-6, Supporting Figures 1-3, and Table 2 and 3 (original Table 1 and 2).
- We created Table 1, Supporting Figure 4, Supporting File 1, and Supporting Table 1.
- We added “Reference 15”.

**Reviewer #1**

**Comment 1)**

Some patients to whom administrated meclizine hydrochloride shows higher concentration than other patients in both of fed and non-fed situations (MEC-01 and MEC-11). Although authors also showed body weight normalized concentration, some patient (MEC-01, 02, 08,10,11) still showed higher concentration compared to other patient. Reviewer wonders why these difference in concentration among the patients occurred and is afraid that these difference might cause the difference of growth promoting effects among patients. Reviewer speculates that the difference in age, sex, sexual maturity status (especially in female because puberty comes earlier in female than in male), height, degree of obesity might cause difference of concentration. In this meaning, age, sex, height SD score, BMI and BMI-SD score, body surface area and Tanner stage of each patient should be shown and why these difference in concentration occurred should be discussed.

**Response 1)**

Thank you for your valuable questions. We created a new “Table 1” that contains subject baseline characteristics. According to your suggestions, we analyzed the variables correlated with differences in plasma concentration of meclizine among subjects, but there were no correlations between C_max_ of meclizine and each variable, including age, sex, height SD score, BMI, body surface area, Tanner staging, and weight in univariate analysis. Since this additional and preliminary analysis had not been described in the study protocol and therefore the results were not included in the revised text. We discuss why these differences in plasma concentration occurred in the second paragraph of Discussion.

**Correction 1)**

- See Table 1.
- Line 159-160
- Line 164-169
- Line 275-280

**Comment 2)**

Reviewer also wonders how the concentration in each patient during 14 days administration was. Is there any difference in concentration among the patient? Please show the pharmacokinetics of each patient during 14 days administration

**Response 2)**

We showed simulated plasma concentration of meclizine during 14 days from MEC-01 through MEC-06, and from MEC-07 through ME-11 in S1A File and S1B File, respectively. We also simulated the PK of meclizine after the 14th day administration using the elimination rate constant between 10 and 24 hours in “once a day group” and 14 and 24 hours in “twice a day group” in all subjects (S4A Fig and S4B Fig). In addition, we demonstrated PK parameters in S1 Table. Coefficient of variant for C_max_ of meclizine among subjects was moderate and reasonable for human PK analysis.

**Correction 2)**

- See Supporting Files 1A and 1B, Supporting Figures 4A and 4B, and Supporting Table 1.
- Line 131
- Line 284-289

**Reviewer #2**

**Comment 1)**

Provide details of how the simulation was conducted with enough granularity that would allow one to replicate the method.

**Response 1)**

We added description of the superposition method in addition to the original manuscript.

**Correction 1)**

- Line 133-134

**Comment 2)**

Mean is the standard terminology for average. Provide a measure of the distribution of the ages, weights and height. Consider including or substituting body mass index for weight and height. Since the sample size is small summarizing the data with medians and interquartile ranges may be more appropriate.

**Response 2)**

Thank you for your important points. We revised according to your suggestions.

**Correction 2)**

- Line 160-169

**Comment 3)**
Include the standard table 1 containing a summary of subject baseline characteristics separated by once or twice a day drug administration schedule.

**Response 3)**

We created a new Table 1 according to your suggestions.

**Correction 3)**

- See Table 1.

**Comment 4)**
Site a reference for calculating the PK AUC.

**Response 4)**

We added “Reference 15”. As a result, following Reference numbers (15-20) were changed in order (16-21).

**Correction 4)**

- See Reference 15.

**Comment 5)**

Consider changing "population" to "analysis."

**Response 5)**

We changed according to your suggestions.

**Correction 5)**

- Line 147, 149

**Comment 6)**

Indicate the statistical approach used for calculating the 95% confidence intervals.

**Response 6)**

We described statistical approach for calculating the 95% CI according to your suggestions.

**Correction 6)**

- Line 147-148

**Comment 7)**

Typographical error: signed
**Response 7)**

Thank you for your point. We revised.
**Correction 7)**

- Line 157

**Comment 8)**

Cite the statistical software used for the analysis.
**Response 8)**

We described the statistical software in the revised manuscript.
**Correction 8)**

- Line 126-127

**Comment 9)**

Indicate the summary values included inside the parenthesis.
**Response 9)**

We added “range” according to your suggestions.
**Correction 9)**

- Line 202, 203, 205, 206, 208, 210, 212, 213

**Comment 10)**

Provide a measure of dispersion for the time.

**Response 10)**

We described dispersion for time of T_max_ according to your suggestions.

**Correction 10)**

- Line 203, 208, 210

**Comment 11)**

Define all abbreviations in table 2. Standard deviation is typically SD.
**Response 11)**

Thank you for your point. We added an abbreviation “SD: standard deviation” in new “Table 2” and “Table 3”.
**Correction 11)**

- See Table 2 and Table 3.

**Comment 12)**

For better clarity, on each figure label the y-axis with an indication of the concentration of meclizine and the time-frame.
**Response 12)**

The y-axis label of “concentration of meclizine” was provided in all previous Figures (Figures 2-6, Supporting Figures 1-3) and new Figures (Supporting Figure 4, Supporting File 1).
**Correction 12)**

- See Figures 2-6, Supporting Figures 1-4, and Supporting File 1.

**Comment 13)**

State and provide justification for the study's target sample size.
**Response 13)**

Although this is an exploratory study and not subject to a formal sample size calculation, we decided the sample size of 6 subjects per group according to similar human PK studies.
**Correction 13)**

- Line 88-91

**Others)**

- We also added supporting information of S4 Fig, S1 Table, and S1 File at the end of Supporting information (Line 404-411).
