# Supplementary material for: Pharmacokinetics and safety after once and twice a day doses of meclizine hydrochloride administered to children with achondroplasia
Source: PLoS One. 2020 Apr 13;15(4):e0229639. doi: 10.1371/journal.pone.0229639 (PMC7153885; doi:10.1371/journal.pone.0229639)
Supplement: Attachment — Submitted filename: Response to Reviewers.docx [file pone.0229639.s010.docx]

- Followings are point-by-point responses to the comments of the editor and the reviewer.
- The sentences and words we changed are written in red in the revised manuscript with track changes.
- Line numbers noted below refer to the revised manuscript.
- We changed Table 1 according to the reviewer’s suggestion.

**Additional Editor Comments)**

The general statement on safety and tolerance in the abstract is based on a very low number of cases as phase 1 studies are not designed to assess safety, to that the editor suggest to adapt this sentence somewhat in the abstract.

**Response)**

Agree with the editor’s comment, we revised the last sentence of abstract and conclusions.

**Correction)**

Line 17-19:

Although higher drug exposure was confirmed in ACH children compared to adults, a single administration of meclizine seemed to be well tolerated.

Line 317-319:

Oral administration of meclizine once a day or twice a day seemed to be safe and well tolerated with no serious adverse events in ACH children.

**Comments from reviewer 1)**

The manuscript is well revised according to the reviewer’s suggestion and critique except for the addition of BMI-SD score to the Table 1. Average of BMI during child period differs according to the age and sex in contrast to the adult whose standard level of BMI is same independent of age and sex. So, it is not appropriate to show the median value among patients of different age and sex in result section (line 165〜166). The BMI-SD score of each patient should be calculated by using the growth evaluation file for children created by the Japanese society of pediatric endocrinology

(available at <http://jspe.umin.jp/medical/chart_dl.html>) and add the each value in Table 1.

**Response)**

Thank you very much for your valuable comments. I totally agree with you in regard to children’s BMI. We re-calculated data from subject baseline characteristics using Excel-based Clinical Tools for Growth Evaluation of Children created by the Japanese Society of Pediatric Endocrinology. We added the BMD-SDS in Table 1. We also deleted the sentence regarding median value of BMI, and instead described the BMI-SDS in the text.

**Correction)**

Line 164-166:

The body mass index-standard deviation score (BMI-SDS) ranged from 0.81 to 2.40 in “once a day group” and from 1.40 to 3.36 in “twice a day group”.

Line 176-177: Table 1 was re-created.
